# Implementation of a multi-level evaluation strategy: a case study on a program for international medical graduates

**DOI:** 10.3352/jeehp.2011.8.13

**Published:** 2011-12-17

**Authors:** Debra Nestel, Melanie Regan, Priyanga Vijayakumar, Irum Sunderji, Cathy Haigh, Cathy Smith, Alistair Wright

**Affiliations:** 1 Gippsland Medical School, School of Rural Health, Monash University, Churchill, Australia.; 2 West Gippsland Healthcare Group, Warragul, Australia.; 3 Imperial College London, London, UK.; 4 Gippsland Regional Clinical School, School of Rural Health, Monash University, Traralgon, Australia.; 5 Department of Family and Community Medicine, University of Toronto, Toronto, Canada.; 6 West Gippsland Hospital, Warragul, Australia.

**Keywords:** Medical education, Educational measurement, Medical students

## Abstract

Evaluation of educational interventions is often focused on immediate and/or short-term metrics associated with knowledge and/or skills acquisition. We developed an educational intervention to support international medical graduates working in rural Victoria. We wanted an evaluation strategy that included participants' reactions and considered transfer of learning to the workplace and retention of learning. However, with participants in distributed locations and limited program resources, this was likely to prove challenging. Elsewhere, we have reported the outcomes of this evaluation. In this educational development report, we describe our evaluation strategy as a case study, its underpinning theoretical framework, the strategy, and its benefits and challenges. The strategy sought to address issues of program structure, process, and outcomes. We used a modified version of Kirkpatrick's model as a framework to map our evaluation of participants' experiences, acquisition of knowledge and skills, and their application in the workplace. The predominant benefit was that most of the evaluation instruments allowed for personalization of the program. The baseline instruments provided a broad view of participants' expectations, needs, and current perspective on their role. Immediate evaluation instruments allowed ongoing tailoring of the program to meet learning needs. Intermediate evaluations facilitated insight on the transfer of learning. The principal challenge related to the resource intensive nature of the evaluation strategy. A dedicated program administrator was required to manage data collection. Although resource-intensive, we recommend baseline, immediate, and intermediate data collection points, with multi-source feedback being especially illuminating. We believe our experiences may be valuable to faculty involved in program evaluations.

## INTRODUCTION

Evaluation is an essential step in curriculum or program development. However, evaluation is often not given prominence during program development, as resources are directed towards implementation. There are benefits associated with evaluation strategies that evolve contemporaneously with program development. These include a clear focus on measurable program outcomes, and an educational design that may promote learning (e.g., deep levels of participant reflection) and can be scheduled as part of the program.

The program evaluation literature has extensively documented many approaches [1-8]. Program evaluation is essential for quality assurance. We adopted a 'traditional' approach to program evaluation that measures structure, process, and outcomes. Examples of 'structural' elements include the content of the program, the number and timing of sessions, physical infrastructure, demographics, and expertise of the faculty. 'Process' elements refer to the usefulness or value of the educational methods and provide insight into faculty and participant reactions to specific sessions and the overall program. 'Outcome' elements refer to changes in participants as a consequence of participating in the program.

In this case study, we describe the development and implementation of the evaluation strategy for a program designed to support the international medical graduates (IMGs) working in rural Victoria, Australia. There are shortages of doctors working in rural practice and IMGs make a substantial contribution to healthcare services. Rural locations are often the first appointment for IMGs in Australia [9-11]. Orientation to the healthcare system is critical but often overlooked. We developed a program - Gippsland Inspiring Professional Standards for International Experts (GIPSIE) to support IMGs working in rural Victoria. Elsewhere, we describe the GIPSIE program and the results of the evaluation [12]. We have summarized key elements of the program in Appendix 1. The GIPSIE program comprised a weekend workshop and four subsequent evening sessions over three months. Simulation-based training was a prominent theme and addressed clinical knowledge, attitudes, and skills, and included a range of activities (e.g., procedural skills training with a part-task trainer, management of the acutely ill patient with manikins, patient assessment skills with simulated patients, etc.). Diverse clinical communication skills were explored (e.g., teamwork, handover, telephone, critical information, etc.). Audiovisual review of performance was enabled through the use of video playback in small groups and later for individual IMGs on iPod Nano devices. GIPSIE was underpinned by a website offering diverse learning resources. Content experts were invited to lead sessions that integrated knowledge and skills reflecting local practice.

GIPSIE had three lead academic faculty (AW, MR, DN) supported by several other academics (including CH, CS), clinicians, and an administrator. Seventeen participants entered the GIPSIE program, which was implemented in 2008 and 2009. Fifteen participants completed GIPSIE and rated the program highly, especially the simulation-based activities with feedback and later audiovisual review on iPods and the GIPSIE website. However, suggestions were made for improving several aspects of the program. Participants reported increased knowledge, skills, and professionalism after the program. Although overall multi-source feedback (MSF) scores showed no statistically significant changes, there were positive directional changes for three items: technical, teaching, and communication skills. These developments were also supported by qualitative comments. Learning was reported to be sustained three months after the program.

In this case study on educational development, we describe the development and implementation process of the evaluation, along with the benefits, and challenges of educational development with the goal of sharing our experiences of the process rather than the outcome of this approach to evaluation.

## MEASURES OF PROGRAM IMPACT

Kirkpatrick [13] developed a 4-level model for evaluating vocational/training programs. The different levels explore trainees' reactions, learning, behavioural changes, and any resulting change in organizational practice. Kirkpatrick's original model implied that all levels are recommended for full and meaningful evaluation of learning. Barr et al. [14] has adapted the original model. The adaptation reveals a 6-level model partly contextualized to healthcare. Appendix 2 illustrates the levels of evaluation, what is measured, examples of evaluation methods, and relevance and practicality [14]. The evaluation methods increase in complexity by level.

We had several goals in the evaluation of GIPSIE. Using the adapted version of Kirkpatrick's model of training impact (Appendix 2), we wanted to access as many levels as possible within our resources. We also wanted to address retention of learning, which is often omitted from training evaluations [15]. That is, we wanted to design an evaluation strategy that would elicit development in trainees' knowledge, attitudes, and skills and detect sustained changes in clinical practice. Here we outline the evaluation strategy and its challenges.

## EVALUATION INSTRUMENTS, DATA COLLECTION, AND ANALYSIS

There were eight instruments in the evaluation strategy and these are listed in Appendix 3. We have divided the time frame for data collection into three stages - baseline data collected prior to participants starting the program, immediate response to the program (including participant reactions) collected during the program, and finally, intermediate-term response to the program when data was collected at least at least three months after the program. All GIPSIE participants were invited to participate in each evaluation activity.

### Baseline data

Baseline data was collected in order to do the following: gain insight to our diverse participants, use this data to ensure a tailored educational program, and have a basis on which to compare outcome data.

#### Instrument 1: Demographics and experience of living and working in Gippsland (Pre-program)

Participants completed a paper-based survey recording age, sex, experience of living and working in Gippsland, career goals, and experience with a range of educational methods. Responses included ratings of satisfaction and free text responses. The survey content was derived from our reading of the literature and issues we considered relevant to our region.

#### Instrument 2: Baseline learning needs analysis (LNA) (Pre-program)

Prior to commencing the program, participants were sent a paper-based form and asked to identify their expectations and learning goals for the GIPSIE program. Responses were in a free text format. The individual and collated content of the LNA were used to adjust the program content and personalize learning. The participants reviewed their LNA during and on completion of the program.

#### Instrument 3: MSF (Pre-program)

The main outcome measure consisted of MSF (pre- and post-program). This is also known as peer assessment or 360 degree feedback. We used a validated instrument designed for workplace-based assessments that is easily integrated with clinical practice [16]. Each IMG was asked to nominate up to twelve colleagues to make judgments on sixteen facets of clinical practice. A six-point scale was provided to reflect level of competence. We also asked participants to self-assess using this form, so that they could build a picture of how they see themselves compared with others.

The process for collecting MSF data is presented in Appendix 4. MSF assessments were completed before and then three months after the program. Assessor identifiers were removed from the collated results provided to the participants.

## IMMEDIATE RESPONSE TO THE PROGRAM

This data was collected to capture participants' experiences of GIPSIE including their perception of changes in knowledge and skills and the usefulness of the educational methods.

### Instrument 4: Workshop evaluation (Weekend workshop)

After the weekend workshop, the participants were given a paper-based form and asked to rate the degree to which they met each prescriptive learning objective (1="not at all met" to 6="completely met") and the educational methods (1="not at all helpful" to 6="completely helpful"). Participants were also asked to identify what worked well and what needed to be improved.

### Instrument 5: End of session evaluations (4 x evening sessions)

Immediately after each evening session, participants were given a paper-based form and asked to rate the degree to which they met prescriptive learning objectives and the educational methods using the same scale described above. Participants identified what worked well and what needed to be improved. We also asked participants to record up to five things they learned in each session providing us with insight into what they valued and what might have been new to them.

## INTERMEDIATE-TERM RESPONSE TO THE PROGRAM (THREE MONTHS AFTER THE PROGRAM)

This data was compared with baseline data to measure the true impact of the GIPSIE program.

### Instrument 6: Telephone interview (Post-program)

A topic guide was used to explore participants' experiences of the program and the impact of those experiences on their work. The topic guide content was developed by program faculty to reflect GIPSIE goals and participant perceptions of the program content and educational methods. Detailed notes were made during the telephone interviews scheduled at a time to meet the needs of participants. These notes were read back to each participant as a process of validation. Some verbatim statements were recorded.

### Instrument 7: GIPSIE website evaluation (Post-program)

User access information was recorded and collated. Participation in online quizzes and other web-based learning activities (e.g., bulletin board) were monitored through frequency of log-in, time online, and number of contributions.

### Instrument 8: MSF or Peer Assessment Tool (PAT) (Post-program)

See instrument 3. Data from individuals was presented in a collated form so that they could monitor their progress from program commencement to completion. We used overall summary data to measure the impact of the GIPSIE program on participants' performance.

The alignment of the evaluation strategy with Kirkpatrick is illustrated in Appendix 2. Baseline data was essential to identify gains post-program so instruments 1, 2, and 3 do not appear in the table, although they are critical to the process. Instruments 4, 5, 6, and 7 explored participant reactions at different points in time (Level 1). Instruments 6 (self-report) and 8 (MSF) provide insight to gains that were assessed after the program was finished, in knowledge (Level 2) and application of learning during and after the program (Level 3). The impact of participants on the clinical environment (Level 4) was intended to be captured by Instrument 8 (MSF). It was not possible for us to address benefits to patients/clients (Level 5) within our resources.

## DATA ANALYSIS

Quantitative data was entered into SPSS ver 18.0 (SPSS Inc., Chicago, IL, USA) for analysis. Descriptive statistics were used to summarize the data. The relatively small sample meant that we used non-parametric statistics. Individual differences pre- and post-program were identified using the Wilcoxon signed rank test. Statistical significance was established at p<0.05.

Qualitative data (free text comments and telephone interview data) were thematically analysed. Themes were identified independently and then agreement negotiated by the researchers (DN, AW, CH). An external evaluator (CS) reviewed de-identified data to ensure rigorous evaluation.

## BENEFITS AND CHALLENGES ASSOCIATED WITH INSTRUMENTS

In this section we identify benefits and challenges of the instruments as we experienced them.

### Instrument 1: Demographics and experience of Gippsland

The benefits of this approach included the ease with which data was collected. Participants readily shared their experiences. Collated data was used in an early session of the program ensuring personalized content. Participants appeared to value this approach, and it provided a platform to share both the highs and lows of living and working in Gippsland. By exploring positives and negatives, we conveyed to participants that we wanted to hear all views. Collection and analysis of data was relatively easy. There were no significant challenges with this instrument except ensuring that individual participants' personal experiences were not revealed without their permission. Sensitive questioning and prompting provided opportunities for further elaboration of relevant information from participants themselves.

### Instrument 2: Baseline learning needs forms

There were several benefits to using this instrument. The most obvious was that participants were encouraged to think deeply about what they wanted to achieve. It also provided us with insight into participants' perceptions of what they thought GIPSIE might be able to address. Learning needs outside the scope of GIPSIE could be clarified at the outset, an important aspect of matching program objectives with participant expectations. Data was easily recorded. The principal challenge (or weakness) was the quality of the information participants provided. On the form, we gave examples of learning needs in order to illustrate how they might be described. Most participants then reported issues similar to the examples we provided. However, some participants provided additional examples and on questioning, the needs appeared genuine.

### Instruments 3 & 8: MSF

Instruments 3 & 8 are same but taken at different times. The main benefit of this instrument at the time of program commencement was that we were able to gain insight to the participants as their colleagues perceived them. Additionally, it conveyed to the participants' colleagues that they were enrolled in a prescribing training program. Although the numerical ratings were interesting, the free text comments were often more helpful, especially when they were detailed. However, the process of collecting the data was highly resource intensive. We collected the data prior to our personally meeting the program participants.

Some participants found it difficult to identify more than eight assessors because they had a relatively short work exposure or worked in small organizations where they were not well known to colleagues outside their unit. The process of collecting the assessor forms required significant follow up and so a designated program manager was required. Despite these challenges, our response rates were satisfactory. Each participant had between two and eight returns at baseline with six the modal return number. After GIPSIE, each participant had five to nine MSF returns with six the modal value. Respondents seemed highly engaged in supporting their IMG colleagues.

We asked IMGs to self assess using this instrument, which provided valuable insight to the participants as to how they viewed themselves in relation to their peers. Although there are issues associated with self-report, participants found the process insightful and sometimes confronting. We had to ensure that participants were supported in making sense of this data, which again, was labour-intensive but highly valued by participants as a learning experience.

### Instruments 4 & 5: Workshop evaluation & End of session evaluations

The benefit of these evaluations was that we received immediate insight into participants' experiences of the program. This helped us adjust subsequent learning objectives and educational methods. For example, in some educational methods, we needed to invest more time in orienting participants to their use (e.g., GIPSIE website questionnaires). Because the evaluation data was collected immediately after the participants' experiences, we were able to adjust subsequent teaching methods. The challenges were associated with participants responding uncritically. Given the relatively small number of GIPSIE participants, there may have been a reluctance to share true feelings, especially if they were critical of the program. We tried to ensure that the completion of forms was a private event and that forms were returned anonymously.

### Instrument 6: Telephone interview

There were several benefits to this method, including the highly personalized nature of data collection. Although participants might have provided what they considered were 'socially desirable' responses, we felt reasonably confident that participants spoke quite freely. The participants seemed to be authentic in their responses. There were very few criticisms. They appreciated the attention and value we placed on their feedback. The challenges were again human-resource related because the interviews were time consuming and there were difficulties in scheduling. We also could not always be certain of where the participants chose to receive the telephone call. The settings might have impeded their freedom to share experiences.

### Instrument 7: GIPSIE website evaluation

Although we planned this collection of data, we did not use it in the final evaluation report. This was mainly associated with the 'remote' management of the GIPSIE website and the relatively small numbers of participants. That is, the website management was commissioned externally, and this seemed to create some communication challenges. Some participants were also very slow to start using the GIPSIE website and with the small cohort size, we were confident that the participants' self-reporting was adequate to meet our evaluation needs.

## CONCLUSION

In order to evaluate the impact of a training program, a carefully planned and resourced strategy is essential. In health professional training, our goal is to ultimately improve the health services offered to patients. However, their direct involvement in evaluation is challenging. Further, programs are often offered by those distant to the workplaces of trainees. Ethical clearances make it difficult for the systematic collection of patient data.

In the project, we sought to implement an evaluation strategy that addressed most levels of the modified Kirkpatrick framework.

Based on our experience, we make the following recommendations:

1. Encourage broad stakeholder involvement in the development of the strategy (e.g., inclusion of Gippsland-based IMGs and lay representatives).
2. Allocate adequate resourcing of administrative support, especially for MSF and booking telephone interviews.
3. Incorporate evaluation data into educational content and process. That is, schedule evaluation activities as part of the curriculum. Use data collected to engage participants in a personalized program while ensuring relevance.
4. If using MSF, then provide clear instructions to participants and assessors to minimize the encroachment on their time. Indicate that free text comments are highly valued if contextualized. Offer reassurance about confidentiality to assessors. Offer reassurance to participants that the results will not be used in any way to influence their employment with their health service.
5. Incorporate participant feedback into ongoing program refinement and delivery to allow for personalization of education strategies as well as clarification of program objectives.
6. Ensure externally commissioned contractual work is clearly articulated and include progress reports.
