# Supplementary material for: Implementation of a multi-level evaluation strategy: a case study on a program for international medical graduates
Source: J Educ Eval Health Prof. 2011 Dec 17;8:13. doi: 10.3352/jeehp.2011.8.13 (PMC3258549; doi:10.3352/jeehp.2011.8.13)
Supplement: Supplementary file 1 [file jeehp-8-13-s001.pdf]

Appendix 1. Key components of the GIPSIE program

Preparation (one month in advance of the program commencement)
- Participants nominate assessors for multi-source feedback (MSF)
- Program administrator commences data collection
- Individual participants complete learning needs analysis (LNA)
- Collation of MSF and LNA data for individuals (learning focus) and summarily (program evaluation)

Two-day weekend workshop
- Individual participants set learning goals
- Educational methods - Discussion-based activities; Experiential activities (including simulations); Web-based activities
- Participants complete written post-workshop evaluations

Four evening sessions (over two months)
- Educational methods - Reflect on learning between sessions; Presentation and discussion of weekly topic; Experiential activities (including simulations); Web-based activities
- Participants complete written post-session evaluations

Workplace observations (at two months)
- Individual participants observed in the workplace by program faculty who used semi-structured observation guides
- Educational methods – Focused discussion on progress, challenges and areas for development; Plan future learning

Post-program evaluation (Three months after the program finishes)
- Telephone interviews conducted by program faculty
- Program administrator commences MSF data collection

GIPSIE: Gippsland Inspiring Professional Standards for International Experts.

Appendix 2. Modified Kirkpatrick levels of evaluation (after Barr et al. [14], 2000)

Level	Evaluation type (what is measured)	Evaluation description and characteristics	GIPSIE evaluation instruments*
1	Participant reaction	Reaction evaluation is how the participants felt about the training or learning experience	4, 5, 6, 7
2	Learning	Learning evaluation is the measurement of the increase in knowledge - before and after the intervention	6, 8
3	Behaviour	Behaviour evaluation is the extent of applied learning back in the clinical setting - implementation	6, 8
4	Results	Results evaluation is the effect on the environment by the trainee	8
5	Benefits to patient/clients	Any improvement in the health and well being of patients as a direct result of an educational program	Not measured

GIPSIE: Gippsland Inspiring Professional Standards for International Experts.
*Instruments 1, 2 & 3 were used to establish baseline data in order to make sense of post-program data.

Appendix 3. Instruments in the evaluation (compressed for presentation)

Instrument 1: Demographics and experience of living and working in Gippsland

1. How old are you?
2. What sex are you? Male/Female
3. What is your country of birth?
4. What is your nationality?
5. What language/s do you speak fluently?
6. What language do you predominantly speak at home?
7. How many years have you been in Australia?
8. How many years have you been in Gippsland?
9. Why did you choose to come to Gippsland?
10. What do you enjoy about Gippsland?
11. What do you dislike about Gippsland?
12. Do you intend to stay in Gippsland?
    a. If yes, why?
    b. If no, why not? Where do you think you will go?
13. What professional qualifications do you have?
    Please list and record the university and country of award

Qualification	University/College	Country

14. To what extent do you enjoy your workplace?

Not at all					Completely
1	2	3	4	5	6

15. To what extent do you think you are supported as an international medical graduate in your workplace? (Circle one)

Not at all					Completely
1	2	3	4	5	6

16. How could you be better supported in your workplace?
17. Have you taken any educational programs designed to support international medical graduates? Yes/No
    If yes, please list and indicate if it was helpful to you.
18. Have you used any of the following simulation-based activities for learning?
    a. Part task trainers Yes/No
    b. Manikins Yes/No
    c. Simulated patients Yes/No
19. Have you ever been the focus of multi-source feedback? Yes/No
    This is an assessment process that is completed by your work colleagues who make judgments about your abilities so you can build up a picture of how others see you. It is sometimes called 360 degree feedback.
20. Have you ever completed a learning needs analysis form? Yes/No
    This is a form designed to help you identify your own learning needs so that a program can be designed to help you meet the needs and goals that you think are most important.
21. Have you ever reviewed yourself on videotape performing a professional activity? (e.g., interviewing a patient) Yes/No

Instrument 2: Learning needs analysis

1. What are you expecting to learn in the Gippsland Inspiring Professional Standards for International Experts (GIPSIE) program?
2. With respect to your current clinical practice, what areas do you feel most confident with?
3. With respect to your current clinical practice, what areas do you think you most need to develop?
4. Think about situations in which you learn most effectively. What are the characteristics of those situations?

Based on your Self mini-PAT (Peer Assessment Tool or Multi-source feedback form) and your answers above, set yourself five learning goals for the GIPSIE program.
Examples:

- To learn to communicate more effectively with colleagues, especially phoning consultants about new admissions.
- To improve my knowledge of current management of stroke
- To prepare for the Australian Medical Council examinations

Goal 1
Goal 2
Goal 3
Goal 4
Goal 5

Instrument 3: Multi-source feedback

Doctor's Name:
Assessor's Name:

How do you rate this doctor in their:	Below expectations		Borderline	Meets expectations of safe and competent performance	Above expectations		Unable to comment
	1	2	3	4	5	6	7
**Good Clinical Care**							
1. Ability to diagnose patient problems							
2. Ability to formulate appropriate management plans							
3. Awareness of their own limitations							
4. Ability to respond to psychological aspects of illness							
5. Appropriate use of resources e.g ordering investigations							
**Maintaining Good Medical Practice**							
6. Ability to manage time effectively / prioritise							
7. Technical skills (appropriate to current practice)							
**Teaching and Training, Appraisal and Assessing**							
8. Willingness and effectiveness when teaching/training colleagues							
**Relationship with Patients**							
9. Communication with patients							
10. Communication with carers and/or family							
11. Respect for patients and their right to confidentiality							
**Working with Colleagues**							
12. Verbal communication with colleagues							
13. Written communication with colleagues							
14. Ability to recognise and value the contribution of others							
15. Accessibility / Reliability							
16. Overall, how do you rate this doctor's performance?							
*U/C Please mark this if you have not observed the behaviour and therefore feel unable to comment.							

Anything going especially well?

Please describe any areas that you think you should particularly focus on for development

Instrument 4: Workshop evaluation

Please help to identify the strengths and weaknesses in this program by completing the following evaluation form.

To what extent did you meet the following learning objectives?

		Not at all				Completely	
1	Describe general principles of information giving	1	2	3	4	5	6
2	Outline how these principles fit within a consultation	1	2	3	4	5	6
3	Demonstrate competence in giving information to patients about a procedure	1	2	3	4	5	6
4	Reflect on ways to maintain and develop communication skills for information giving	1	2	3	4	5	6

How effective do you think the following educational techniques were in relation to meeting the learning objectives?

		Not at all				Completely	
5	Session guide	1	2	3	4	5	6
6	Discussion	1	2	3	4	5	6
7	Feedback from simulated patient	1	2	3	4	5	6
8	Feedback from tutor	1	2	3	4	5	6
9	Feedback from peers	1	2	3	4	5	6

Please add further comments here.

Instrument 5: Session evaluation (one example)

Please help to identify the strengths and weaknesses in this program by completing the following evaluation form.

To what extent did you meet the following learning objectives?

		Not at all				Completely	
1	To identify effective patient-centred communication skills	1	2	3	4	5	6
2	To reflect on difficult communication challenges in clinical settings	1	2	3	4	5	6
3	To practice patient-centred interviewing skills in a simulated consultation	1	2	3	4	5	6
4	To demonstrate a logical approach to a clinical problem (infection)	1	2	3	4	5	6
5	To stratify the risk of complications by understanding appropriate tools that identify important elements of a patient's assessment	1	2	3	4	5	6
6	To improve knowledge with respect to specific infections including staphlococcal sepsis and community acquired pneumonia	1	2	3	4	5	6
7	To apply the knowledge gained in the infection lecture to a simulated clinical interaction	1	2	3	4	5	6
8	To reflect on communication challenges in the simulated clinical interaction	1	2	3	4	5	6

How effective do you think the following educational techniques were in relation to meeting the learning objectives?

		Not at all				Completely	
1	Session guide	1	2	3	4	5	6
2	Lecture session on communication	1	2	3	4	5	6
3	Discussion and review of videos of simulated consultations	1	2	3	4	5	6
4	Participating in the simulations	1	2	3	4	5	6
5	Feedback from simulated patients	1	2	3	4	5	6
6	Feedback from tutors on performance in simulation	1	2	3	4	5	6
7	Giving feedback to peers on performance in simulation	1	2	3	4	5	6
8	Receiving feedback from peers on performance in simulation	1	2	3	4	5	6
9	Lecture session on infection	1	2	3	4	5	6

List five things you learned in the session. These are not always 'new' things but the opportunity to revisit things you already knew but see them differently, in more detail, in a new context etc. This process is to help you reflect on what you are getting from the GIPSIE program.

1.
2.
3.
4.
5.

In order to help us evaluate the session, please answer the following questions:
What worked well?
What needs development?

Instrument 6: Telephone interview

The following questions are a guideline for the individual interviews with participants after the GIPSIE program.

1. What thoughts do you have about the GIPSIE program?
2. What was valuable?
3. What was not?
4. What needs to be improved?
5. What could have been added?
6. What could have been left out?
7. What do you think about the use of simulation to help you learn?
8. What do you think about the multi-source feedback to help you learn?
9. What do you think about the GIPSIE website to help you learn?
10. What do you think about working in small groups as you did on the GIPSIE program?
11. Would you recommend this program to others?

Instrument 7: GIPSIE website evaluation

The following activities will be monitored on the GIPSIE website by participant (de-identified).

1. Frequency of log-in
2. Length of time on line
3. Time of day
4. Relative to program meetings
5. Sections of website accessed
   a. Quizzes
   b. Program information (scheduling, etc)
   c. News
   d. Discussion forum
   e. Other

Instrument 8: Multi-source feedback

As for instrument 3

Appendix 4. Instructions for GIPSIE participants

Please read and return to the Gippsland Inspiring Professional Standards for International Experts (GIPSIE) project manager (INSERT NAME) by (INSERT DATE).

Multi-source feedback (MSF) enables you to learn how others see you compared with how you see yourself. It is not always comfortable but it is a powerful way of learning. It is also a way that we can evaluate the impact of the GIPSIE program.

The GMS faculty are experienced teachers who will work with you to help make sense of the feedback constructively. Based on the MSF results and your own learning needs analysis, activities in the GIPSIE program can be adjusted to meet your individual needs (without disclosing details of your feedback to peers or anyone else).

The MSF rating form has 16 items – each item is important in describing the all round performance of doctors.

You are asked to nominate twelve assessors, including their contact details. Selecting people with whom you work closely is important and should include peers, senior doctors, nurses and other health professionals. It is also appropriate to have administrative staff who may not be able to rate all items but that is also acceptable.

You will not know who has given the rating. This will be de-identified.

After nominating your assessors, the project manager will contact the assessors by mail and invite them to complete and return the form. The MSF form is designed to take less than 10 minutes to complete.

On receiving the completed forms, the project manager will enter the results into a database enabling summary results to be produced for each of you. These will be given to you at the beginning of the GIPSIE program. You will develop a learning plan based on the MSF and your own learning needs analysis.

Nominations for multi-source feedback

Try to select at least two people from each role. The more you include the better. Try to obtain a minimum of eight nominations. Please identify people at your current workplace. You need only provide one address, just indicate the best way to contact the nominee in the hospital (e.g., ward).

Name	Role	Work address (ward)
	Senior doctor	
	Senior doctor	
	Senior doctor	
	Peer	
	Peer	
	Peer	
	Nurse	
	Nurse	
	Nurse	
	Other health professional	
	Other health professional	
	Other health professional	
	Administrator	
	Administrator	
	Administrator	

Place of work:
